# Supplementary material for: Açai supplementation (Euterpe oleracea Mart.) attenuates cardiac remodeling after myocardial infarction in rats through different mechanistic pathways
Source: PLoS One. 2022 Mar 4;17(3):e0264854. doi: 10.1371/journal.pone.0264854 (PMC8896726; doi:10.1371/journal.pone.0264854)
Supplement: S1 Table — (DOCX) [file pone.0264854.s001.docx]

**S1 Table. Dose-dependent effects of different doses of açai supplementation.**

|  | P value |
| --- | --- |
| SOD (nmol/mg) | 0.005 |
| GPx (nmol/mg) | 0.014 |
| LH (nmol/mg) | 0.034 |
| MDA (μmol/g) | 0.009 |
| PDH (nmol/g) | 0.001 |
| CS (nmol/g) | 0.043 |
| Complex I (nmol/g) | 0.003 |
| LDH (nmol/g) | <0.001 |
| IL-10 (pg/mg) | <0.001 |
| *ICF (%) | 0.016 |
| TIMP-1 (pg/mg) | <0.001 |

SOD: superoxide dismutase; GPx: glutathione peroxidase; LH: lipid hydroperoxide; MDA: malondialdehyde; PDH: pyruvate dehydrogenase complex, CS: citrate synthase, LDH: lactate dehydrogenase; IL-10: interleukin 10; ICF: interstitial collagen fraction; TIMP-1: tissue inhibitor of metalloproteinase-1. Spearman’s rank correlation coefficient test was performed to compare the dose-dependent effects of açai supplementation in infarcted animals.
