# Supplementary figures and images for: Açai supplementation (Euterpe oleracea Mart.) attenuates cardiac remodeling after myocardial infarction in rats through different mechanistic pathways
Source: PLoS One. 2022 Mar 4;17(3):e0264854. doi: 10.1371/journal.pone.0264854 (PMC8896726; doi:10.1371/journal.pone.0264854)

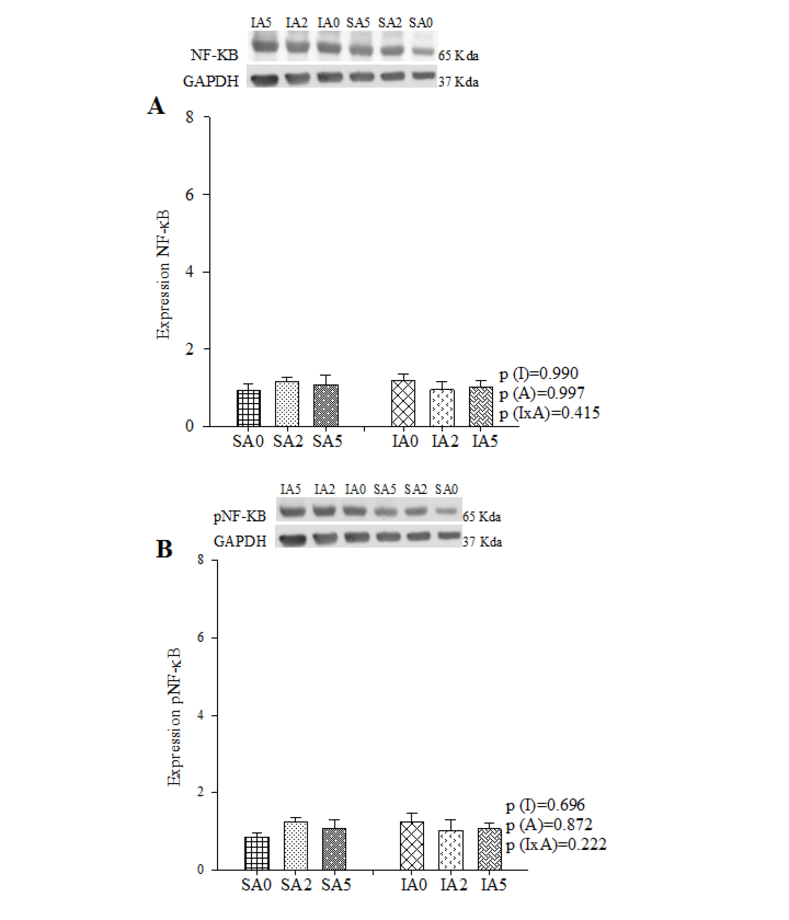

Supplement: S1 Fig — (A) NF-κB total: nuclear factor kappa B, (B) pNF-κB: nuclear factor kappa B: phosphorylated nuclear factor kappa B. Sample size: SA0 = 8; SA2 = 8; SA5 = 6; IA0 = 6; IA2 = 6; IA5 = 7. (TIF) [file pone.0264854.s002.tif]

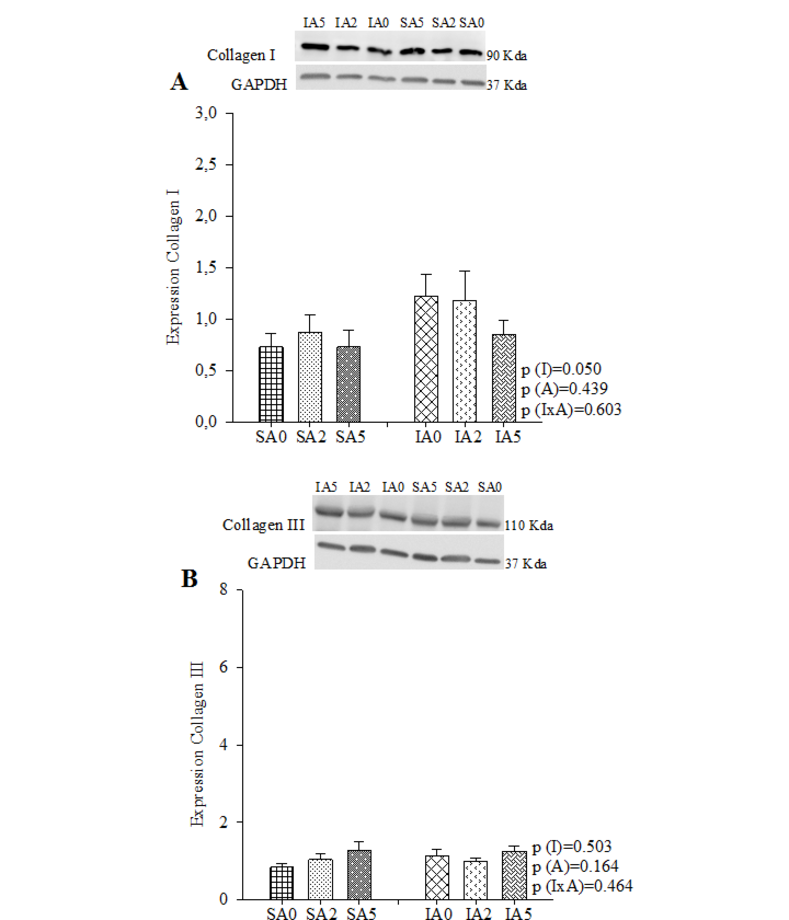

Supplement: S2 Fig — (A) Collagen I. Sample size: SA0 = 8; SA2 = 6; SA5 = 6; IA0 = 6; IA2 = 6; IA5 = 6. (B) Collagen III. Sample size: SA0 = 7; SA2 = 6; SA5 = 6; IA0 = 6; IA2 = 6; IA5 = 6. (TIF) [file pone.0264854.s003.tif]

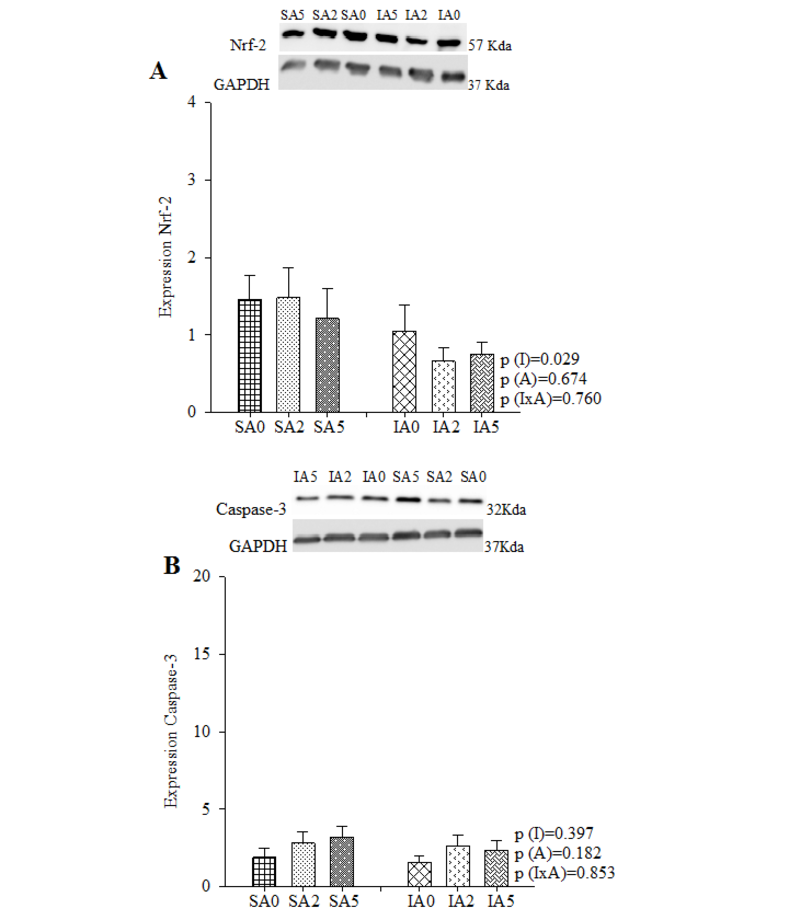

Supplement: S3 Fig — (A) Nrf-2: expression of nuclear factor erythroid-2. Sample size: SA0 = 8; SA2 = 8; SA5 = 7; IA0 = 6; IA2 = 8; IA5 = 8. (B) Caspase-3. Sample size: SA0 = 8; SA2 = 7; SA5 = 8; IA0 = 7; IA2 = 8; IA5 = 7. (TIF) [file pone.0264854.s004.tif]
